# UV-C inactivation of Legionella rubrilucens

**DOI:** 10.3205/dgkh000291

**Published:** 2017-04-10

**Authors:** Julian Schmid, Katharina Hoenes, Monika Rath, Petra Vatter, Martin Hessling

**Affiliations:** 1 Ulm University of Applied Sciences, Ulm, Germany

**Keywords:** Legionella, UV-C radiation, mercury vapor lamp, log-reduction dose, point-of-use disinfection

## Abstract

**Background:** Despite the great health significance of *Legionella*, there is only little information on their UV sensitivity. Besides *Legionella pneumophila* only *L. longbeachae* has been investigated so far.

**Methods:** In this study *L. rubrilucens* has been spread on buffered charcoal yeast extract agar and irradiated with the 254 nm UV-C emission of a mercury vapor lamp. The disinfection success is measured by colony counting after incubation and comparison of the number of colonies on irradiated and unirradiated reference agar plates.

**Results:** The average log-reduction dose is 1.08 mJ/cm^2^ for free *L. rubrilucens*, which is at the lower end of the so far published Legionella log-reduction values, but all three *Legionella* species show similar UV-C sensitivities.

**Conclusion:** The log-reduction dose of legionellae in amoebae has not been investigated, but with the observed high UV-C sensitivity for free *Legionella*, the idea of a future point-of-use disinfection by small UV-C LEDs in water-taps or shower heads appears to be realistic, even if legionellae are more resistant in amoebae.

## Zusammenfassung

**Hintergrund:** Trotz der großen gesundheitlichen Bedeutung von Legionellen gibt es bisher nur wenige Untersuchungen zu ihrer UV-C-Empfindlichkeit. Außer *Legionella pneumophila* wurden bisher nur Ergebnisse für *L. longbeachae* veröffentlicht.

**Material und Methoden:** In der hier vorgestellten Untersuchung wurden Bakterienlösungen mit *L. rubrilucens* auf gepuffertem Aktivkohle-Hefe-Extrakt verteilt und der 254 nm UV-C-Strahlung einer Quecksilberdampflampe ausgesetzt. Der Desinfektionserfolg wird durch die Zählung von Legionellen-Kolonien nach der Bebrütung ermittelt, indem die Anzahl der Kolonien auf bestrahlten Agarplatten mit der Zahl an Kolonien auf unbestrahlten Referenzplatten verglichen wird.

**Ergebnisse:** Die durchschnittlich notwendige UV-C-Strahlungsdosis für eine Log-Stufen-Reduktion beträgt 1,08 mJ/cm^2^ für planktonische *L. rubrilucens*. Dieser Wert liegt im unteren Bereich der 4 bisher veröffentlichten Legionellen-Studien und zeigt aber auch, dass alle 3 bisher untersuchten *Legionella*-Spezies ähnliche UV-C-Empfindlichkeiten aufweisen.

**Schlussfolgerung:** Die UV-C-Empfindlichkeit aller Legionellen-Spezies scheint vergleichbar hoch zu sein. Selbst wenn Legionellen in Amöben etwas unempfindlicher sein sollten, scheint die Idee einer zukünftigen Point-of-Use-Desinfektion sehr realistisch. Kleine UV-C-LEDs in Wasserhähnen und Duschköpfen könnten Legionellen im schnell vorbeiströmenden Wasser signifikant inaktivieren. Der Einsatz einer solchen Technik scheint insbesondere in Gesundheitseinrichtungen wie Krankenhäusern oder Altenheimen sinnvoll.

## Introduction

In 2014 about 7,000 cases of detected Legionnaires’ Disease were reported in the European Union [1] and the number of unreported infections is probably much higher. Legionnaires’ Disease is the most severe form of pneumonia infections caused by Gram-negative *Legionella* spp. The most pathogenic *Legionella* species is *Legionella pneumophila* that was identified in the first documented *Legionella* outbreak in Philadelphia in 1976 [2]. Legionellae infections often occur by inhaling aerosols derived from contaminated water sources. The reservoirs of these Gram-negative bacteria are natural surface water, and especially drinking and cooling water systems [3]. There are different approaches for clearance of legionellae in these man-made water reservoirs but they usually do not show long lasting effects leading to an omnipresent danger of a *Legionella* contamination.

Among the well-known disinfection techniques is the application of hot water with temperatures of about 70°C, but it is technically difficult to reach these high temperatures, especially in long pipe systems with lengths of 10 m and more. Chemical disinfection, e.g. by chlorine compounds, shows a reduced disinfection effect due to the fact that legionellae are often incorporated in biofilms and amoebae that protect the bacterium against direct contact with disinfectants [4]. 

**Figure 1:**
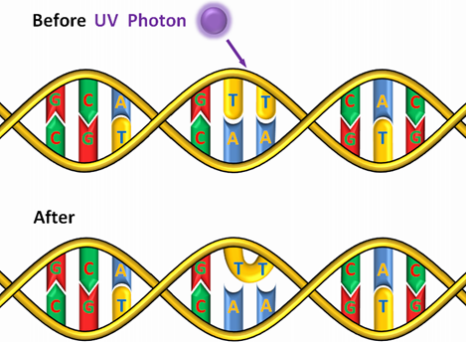
Absorption of a UV-C photon by DNA and formation of a thymine dimer

UV-C radiation was successfully applied for water disinfection already 100 years ago [5]. The radiation is usually generated by mercury vapor lamps, which show strong emission at a wavelength of 254 nm. The UV-C radiation is absorbed by DNA, resulting in the formation of thymine dimers [6] as illustrated in Figure 1 (Fig. 1). This DNA damage hinders gene expression and DNA replication and should lead to the death of the irradiated microorganisms. Unfortunately UV-C disinfection offers no depot effect. Bacteria that survived the irradiation can proliferate again on their way through the water pipe system. Extremely aggravating is the formation of biofilms on the pipe walls that are capable of repeatedly releasing microorganisms including *Legionella* that might get in direct contact with humans without a further disinfection step. 

With the recent developments of UV-C LEDs the idea of a point-of-use disinfection with an UV-C LED directly installed in the water-tap or shower head becomes more realistic. These LEDs are very small and offer only low radiation intensities in the range of up to 90 mW [7], which is about a factor of 100 below strong mercury vapor lamps, but do not contain mercury and offer a longer life time. The UV-C LED radiation power might be sufficient for *Legionella* inactivation, because according to [8], [9], [10], [11] the necessary log-reduction dose for *L. pneumophila* is somewhere between 0.9 and 3.1 mJ/cm^2^ and 1.4 mJ/cm^2^ for *L. longbeachae* [9] – a dose that could be reached within 0.03 s if a 90 mW UV-C LED irradiates an area of 1 cm^2^. 

The intention of this paper is to get more information on necessary log-reduction doses for legionellae by performing experiments on *L. rubrilucens* and compare them with the so far reported log-reduction doses. *L. rubrilucens* was reported as co-pathogen in a case of Legionnaires’ Disease [12] but its UV-C sensitivity has not been investigated so far.

## Materials and methods

**Figure 2:**
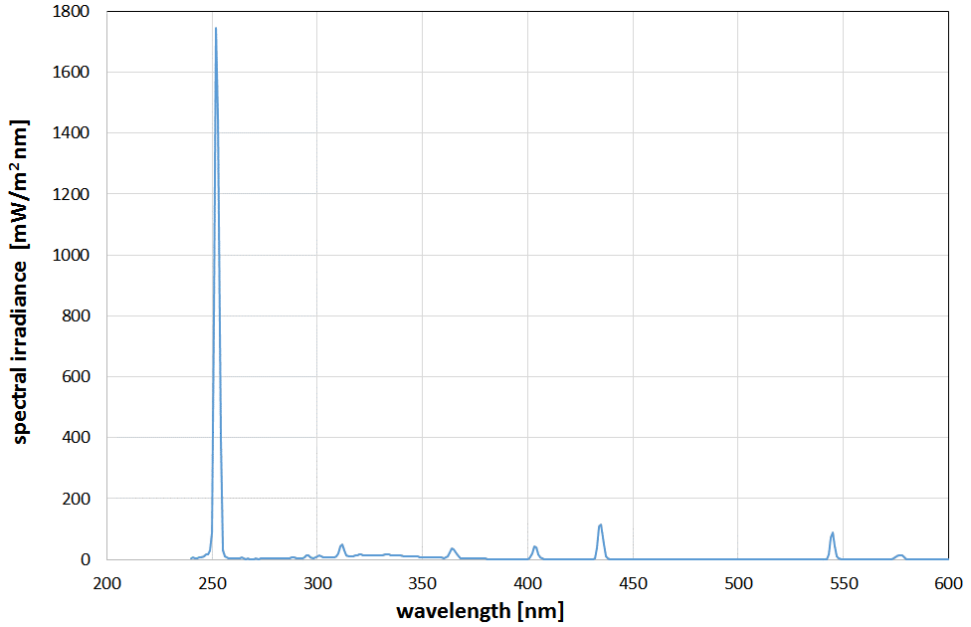
Emission spectrum of the employed low pressure mercury vapor lamp

The UV-C radiation was generated by a low pressure mercury vapor lamp type TUV 8W FAM/10X25BOX of Philips Lighting Holding B.V. (The Netherlands). It offers several emission lines in the UV and visible spectral range, but the emission at 254 nm is by far the strongest as can be seen in Figure 2 (Fig. 2). In a distance of 15 cm the obtained 254 nm irradiance was 0.47 ± 0.05 mW/cm^2^ over an area of 10 x 30 cm^2^. It should be mentioned that the total irradiance, including UV-B, UV-A and visible light is slightly higher with about 0.65 mW/cm^2^ but the disinfection contribution of these longer wavelength emissions to the disinfection result is negligible with about 3% of the impact of the 254 nm emission, calculated with the spectral antimicrobial action spectra values given in [13] for *Escherichia coli*, but it is assumed that other bacteria show a similar behavior. 

The bacteria investigated were *L. rubrilucens* (DSM No. 11884) that were obtained as freeze-dried culture from Deutsche Sammlung von Mikroorganismen und Zellkulturen GmbH (Braunschweig, Germany) and cultivated in buffered yeast extract (BYE) broth [14]. For the UV-C inactivation experiments solid buffered charcoal yeast extract (BCYE) agar [14] was prepared in Petri dishes with a diameter of 90 mm.

Bacterial suspensions of *Legionella* with an optical density of 0.24 at 600 nm, that contained about 2.4 x 10^8^ CFU/ml in preliminary experiments, were further diluted in phosphate-buffered saline to concentrations below 10,000 CFU/ml. 100 µl of this samples were carefully dispensed over the surface of the BCYE agar plate.

**Figure 3:**
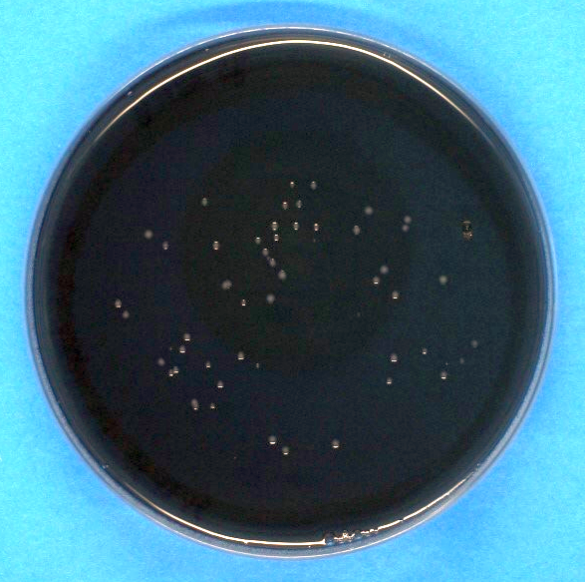
Example of a BCYE agar plate with visible colonies of *Legionella* after incubation

The radiation experiments were performed with different exposure times to achieve different irradiation doses. The maximum irradiation time was 12 s, which resulted in doses of up to 5.6 mJ/cm^2^. Afterwards the agar plates were stored in an incubator at 37°C and high humidity for 4 days before the plates were photographed for better colony counting (see example in Figure 3 (Fig. 3)) and the observed colonies on irradiated agar plates were compared to unirradiated reference plates. This procedure was performed three times with three agar plates for each irradiation dose and each run.

## Results and discussion

**Figure 4:**
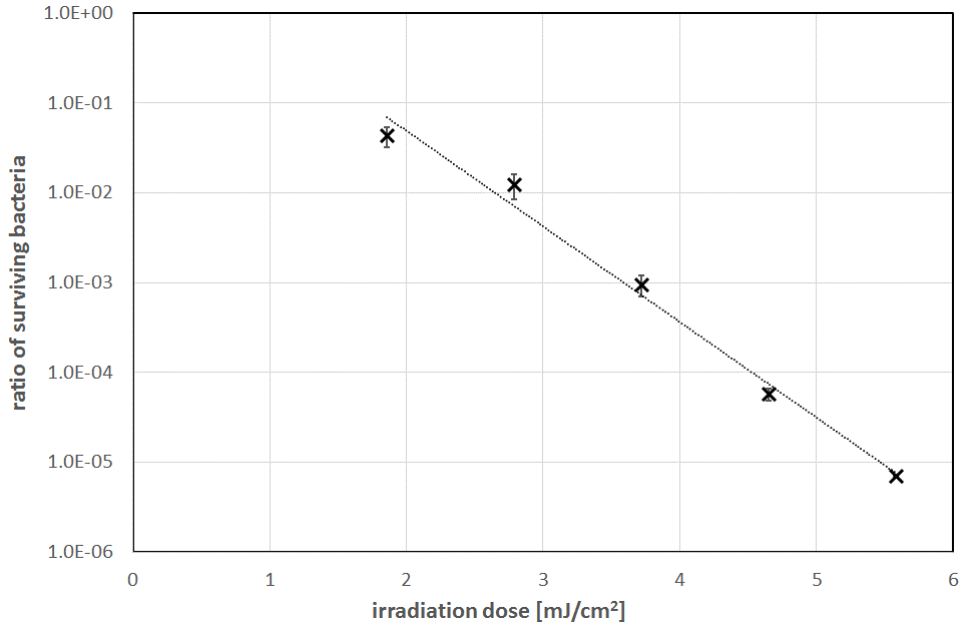
Bacterial reduction for *L. rubrilucens* during UV-C irradiation. Error bars depict the standard deviation of the average of three independent measurements.

The disinfection results are presented in Table 1 (Tab. 1) and Figure 4 (Fig. 4). For higher irradiation doses the measured ratios of surviving bacteria follow a straight line in this half logarithmic diagram, which is equivalent to an exponential decrease for increasing UV-C doses. But this straight line doesn’t pass the expected 100%-point (1.0 E+00) for 0 mJ/cm^2^ irradiation. This well-known phenomenon is called “shoulder effect” [15]. The UV-C sensitivity is lower for lower doses and increases asymptotically for higher doses. Lower sensitivity means a higher necessary irradiation dose to reach a log-reduction (log-reduction: decrease by a factor of ten). The lowest irradiation dose of 1.86 mJ/cm^2^ in Table 1 (Tab. 1) led to 4.3% or 4.3 E-02 surviving bacteria. This is equivalent to a log-reduction dose of 1.36 mJ/cm^2^. But if the calculation is based on the highest applied irradiation dose of 5.58 mJ/cm^2^ and 0.0007% or 7.0 E-06 surviving bacteria the average log-reduction dose for *L. rubrilucens* becomes 1.08 mJ/cm^2^. 

These results are in good agreement with published *L. pneumophila* and *L. longbeachae* log-reduction doses of 0.9 and 1.6 mW/cm^2^ measured by [8], [9], [10]. 

It has to be mentioned that the here applied incubation durations of 4 days at 37°C after irradiation were rather short compared to several other published incubation times. Antopol and Ellner incubated their irradiated samples for 4–5 days at 35°C [8], Oguma et al. for 7 days [10] and Cervero-Arago et al. even for 10 days at 37°C [9]. It is theoretically conceivable that legionellae are still in a viable but nonculturable (VBNC) state [16] after 4 days, and over the course of time could recover by dark repair and photo reactivation mechanisms [17] and subsequently form colonies again. This would reduce the real UV sensitivity or increase the log reduction dose. However, there is no general rule for *Legionella* incubation periods. The international DIN-ISO standard “Water quality – Enumeration of *Legionella*” [18] gives different incubation times depending on the application. In the case of samples, which are not clear for the presence of legionellae, a 7–10 day incubation should be carried out but for confirmation of legionella suspicion samples should be incubated for a maximum of 5 days. The Australian Legionella Control Guidelines indicate that the incubation period may take up to 10 days, but the average is between 5–7 days [19].

It is to be expected that in the presented experiments, no difference in the number of colonies caused by VBNC legionellae would have been determined between 4 and 10 days of incubation. The first reason for this assumption is the observation that legionellae in the VBNC state seem to need amoebae for their recovery [20] that were not present here in any case. The second reason is the fact that even 10 days is a rather short period compared to the 145 days and more, in which legionellae could stay in a VBNC state according to Alleron et al. [21].

Very important is the fact that legionellae in the VBNC state – at least in an guinea pig animal model – do not lead to infections [20]. Therefore, they probably play a subordinate role during the immediate period after irradiation.

So the observed log-reduction dose of about 1.08 mW/cm^2^ for *L. rubrilucens* should not be influenced by the shorter incubation time and the result consolidates the impression that different *Legionella* species show a similar high sensitivity to UV-C radiation. Legionellae in amoebae required almost twice the UV-C dose of free legionellae [9], but this is still a high sensitivity resulting in log-reduction doses that are applicable with UV-C LEDs within fractions of a second. This supports the idea of a future point-of-use water disinfection by small UV-C LEDs that are integrated in water-taps and shower heads and irradiate the pre-passing water at least in healthcare institutions like hospitals or nursing homes.

## Notes

### Acknowledgement

This work was financially supported by the German Federal Ministry of Economics and Technology within the ZIM project “Clean Spring” (grant number KF2186208CR4).

### Competing interests

The authors declare that they have no competing interests.

## Figures and Tables

**Table 1:**
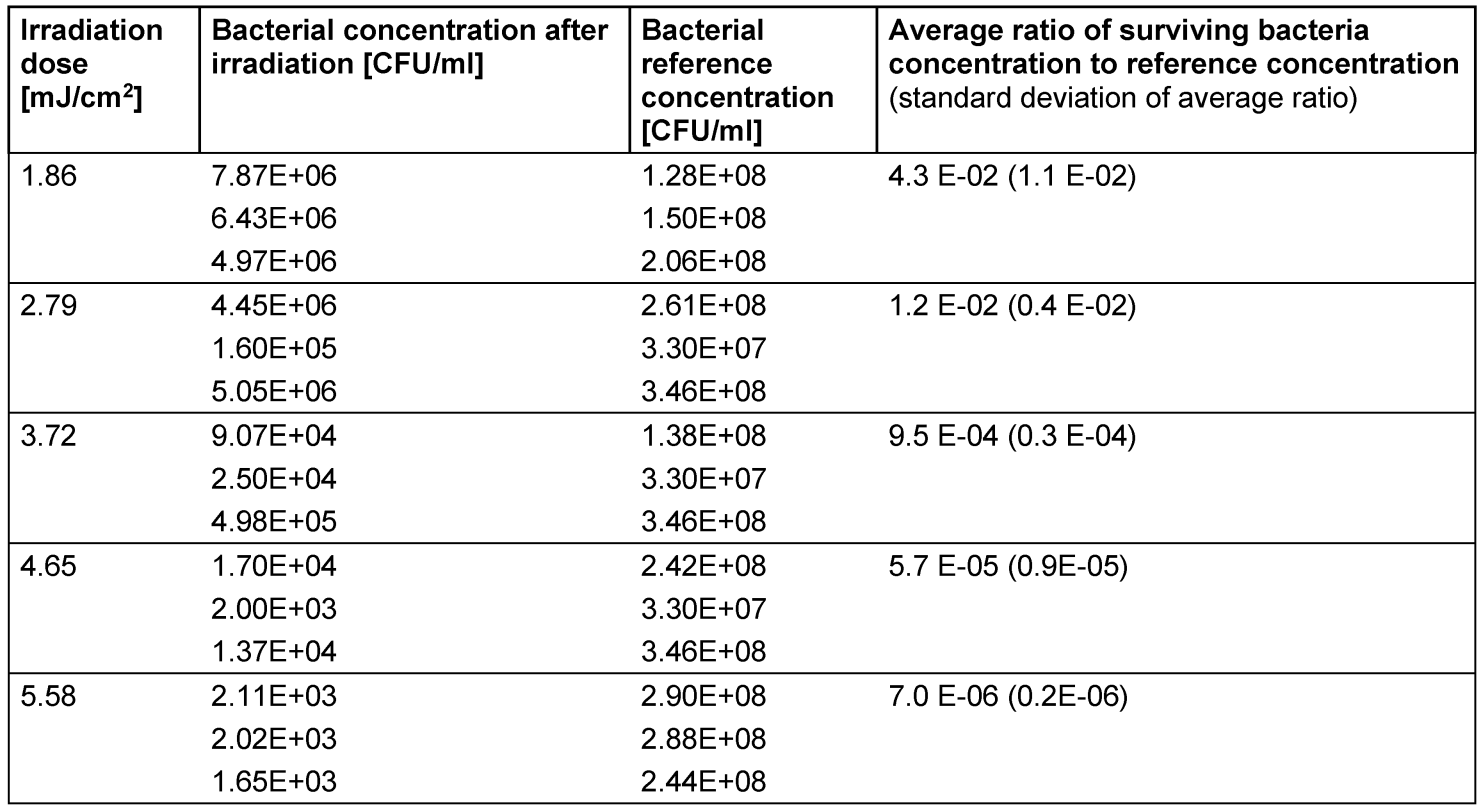
Applied UV-C irradiation doses, average bacterial concentrations after irradiation for each run, average reference concentrations for each run and ratio of surviving bacteria (averaged over all three runs)
